# Uncovering structural variants associated with body weight and obesity risk in labrador retrievers: a genome-wide study

**DOI:** 10.3389/fgene.2023.1235821

**Published:** 2023-09-20

**Authors:** Michal Antkowiak, Maciej Szydlowski

**Affiliations:** Department of Genetics and Animal Breeding, Poznań University of Life Sciences, Poznań, Poland

**Keywords:** labrador retriever, obesity, ALPL, KCTD8, SGSM1, SLC12A6, RYR3, VPS26C

## Abstract

Although obesity in the domestic dog (*Canis lupus* familiaris) is known to decrease well-being and shorten lifespan, the genetic risk variants associated with canine obesity remain largely unknown. In our study, which focused on the obesity-prone Labrador Retriever breed, we conducted a genome-wide analysis to identify structural variants linked to body weight and obesity. Obesity status was based on a 5-point body condition score (BCS) and the obese dog group included all dogs with a BCS of 5, along with dogs with the highest body weight within the BCS 4 group. Data from whole-gene sequencing of fifty dogs, including 28 obese dogs, were bioinformatically analyzed to identify potential structural variants that varied in frequency between obese and healthy dogs. The seven most promising variants were further analyzed by droplet digital PCR in a group of 110 dogs, including 63 obese. Our statistical evidence suggests that common structural mutations in or near six genes, specifically *ALPL*, *KCTD8*, *SGSM1*, *SLC12A6*, *RYR3*, and *VPS26C*, may contribute to the variability observed in body weight and body condition scores among Labrador Retriever dogs. These findings emphasize the need for additional research to validate the associations and explore the specific functions of these genes in relation to canine obesity.

## Introduction

Canine obesity is a significant problem because of its high prevalence and its association with unfavorable physiological conditions and diseases. Obesity is a common phenomenon in countries where dogs are kept as pets, being perhaps as common as human obesity. For example, in Dutch populations, overweight affects forty percent of dogs and obesity another twenty percent, whereas over forty percent of dogs in Beijing are affected by obesity (Courcier et al., 2010; Mao et al., 2013). Obesity is associated with obesity-related metabolic dysfunction (Tvarijonaviciute et al., 2012), orthopaedic disease, breathing difficulties and urinary incontinence, which reduce wellbeing and shorten life expectancy.

As in humans, common canine obesity is polygenic with contribution from a large number of genes, most with small effect size, and a significant environmental influence. In dogs, the contribution of polygenes to the overall variability, traditionally measured by the heritability coefficient, is difficult to estimate due to population fragmentation (both between and within breeds), incomplete pedigree records, and the lack of systematic data collection. Various characteristics of obesity, however, show similarity across mammals, and reliable heritability estimates exist for humans, mice, and pigs. From this point of view, it is believed that obesity is moderately inherited in dogs (Switonski and Mankowska, 2013). A study of Strandberg et al. (2023) presented a comprehensive investigation into the heritability of body weight - a trait influenced by both body size and body fat accumulation. The analysis across 19 breeds estimated the average heritability of body weight to be 51%, with a range of 35%–70%. Moreover, the fact that certain breeds—such Labrador retriever, Golden retriever, Pug, and Beagle—are especially prone to obesity indicates that some polygenes are fixed or very common in these breeds. The observation that Labrador dogs with the lowest birth weights are more likely to become overweight in adulthood can be explained by a genetic correlation between the two traits and indirectly suggests the existence of significant genetic variability related to obesity (Mugnier et al., 2020).

In contrast to human obesity, we know relatively little about the effects of individual genes and markers of obesity (Wallis and Raffan, 2020). From studies on humans and model animals, it appears that the genes implicated in the monogenic type of obesity may also carry mutations that stand out from the polygenic background (*LEP, PCSK1, LEPR, POMC, MC4R, SIM1, NTRK2, BDNF, SH2B1, MRAP2, KSR2, ADCY3*). This applies primarily to genes in the melanocortin pathway. A few effects of individual canine genes have been identified, including *POMC, TNF, MC4R, GPR120 and ADCY3* (Zeng et al., 2014; Miyabe et al., 2015; Mankowska et al., 2016; Raffan et al., 2016; Mankowska et al., 2017; Sypniewski and Szydlowski, 2023). The outcomes of genome-wide association studies (GWAS) pertaining to body mass—an attribute intricately correlated with body fat—imply in a roundabout manner that genetic variations within *ACSL4*, a gene responsible for regulating back fat thickness in pigs, might likewise play a role in the susceptibility to canine obesity (Plassais et al., 2017). In contrast to investigations concerning human obesity, association analyses conducted in canines exhibit diminished statistical power, and the outcomes are frequently lacking validation within separate populations (except *POMC*). Consequently, our capacity is limited to identifying solely those variants that wield substantial influence over obesity-related traits.

The modest headway in pinpointing short variants linked to canine obesity underscores the potential advantages of incorporating an exploration into structural variants (SVs) within obesity research, given their anticipated capacity for exerting more pronounced individual effects. This move is facilitated by progress in computer-aided identification of structural variants based on genome-wide sequencing (Ionita-Laza et al., 2009; Teo et al., 2012). Arguments for the role of structural variants in obesity traits can be found in the discussions on the relationship between copy number variation (CNV) in the *AMY* gene and process of domestication (Axelsson et al., 2013). The first clue that structural variants in genes from the *AMY* family are associated with obesity was found in humans, where some data show correlation between the copy number of the *AMY1* gene, coding for pancreatic amylase, and body mass in children (Mejía-Benítez et al., 2015; León-Mimila et al., 2018), as well as Type 2 diabetes (Liu et al., 2020).

In this study, we search the dog genome for structural variants that may affect body weight and the predisposition of Labrador retriever dogs to obesity. We analyze genome-wide sequencing data from fifty dogs, including obese and nonobese individuals, and use bioinformatic methods to select polymorphic sites that may be significant for obesity-related traits. We validate these sites using the dd-PCR technique in a sample of 110 dogs. We find statistical evidence of a link between six structural variants in or near six protein-coding genes and obesity-related traits. We then justify the need to verify these preliminary associations in a broader study.

## Materials and methods

### Samples and whole-genome sequencing data

We used data on 110 Labrador retriever dogs, collected at four veterinary clinics during routine examinations from 2014 to 2017. All the dog owners gave their consent for participation in the research. The following data were collected: sex, age, body weight, neuter status, and body condition score on a five-point scale. Pedigree data were not collected.

To facilitate statistical analysis, the population was categorized into two groups: nonobese dogs and obese dogs. The nonobese dog group comprised all dogs with a body condition score (BCS) of 3, as well as dogs with the lowest body weight within the BCS 4 group (<33 kg). Conversely, the obese dog group included all dogs with a BCS of 5, along with dogs with the highest body weight within the BCS 4 group (>33 kg). The mean age and body weight were 56.7 months (SD = 33.8) and 36.2 kg (SD = 5.4 kg), respectively. The sample is summarized in Table 1.

**TABLE 1:** Summary of the sample of 110 Labrador retriever dogs used in this study. Presented vales are medians (and range). The records were collected from 2014 to 2017.

Dogs	N	Age (month)	Body weight (kg)
All	110	48 (12–120)	36.0 (25.0–50.0)
Males	46	60 (12–120)	38.0 (31.0–50.0)
Females	64	48 (12–120)	34.0 (25.0–46.0)
Neutered males	7	60 (36–96)	35.0 (32.0–44.0)
Neutered females	16	72 (36–120)	31.1 (26.0–44.0)
BCS = 3	32	36 (12–108)	30.5 (25.0–43.0)
BCS = 4	52	48 (12–120)	36.1 (30.0–48.1)
BCS = 5	26	76.5 (24–108)	41.75 (35.0–50.0)
Nonobese	47	36 (12–120)	32.0 (25.0–40.0)
Obese	63	60 (12–120)	39.0 (32.4–50.0)

Of these 110 dogs, 28 obese and 22 nonobese dogs were subjected to whole-genome sequencing as part of our previous study (Szydlowski and Antkowiak, 2022). FASTQ files are publicly available in the European Nucleotide Archive (PRJEB47658). Detailed information on mapping sequence reads on a reference genome is given in our previous report. Briefly, raw FASTQ files for each sample were aligned with the canine reference genome CanFam3.1 using BWA software (Li and Durbin, 2010). The aligned reads in SAM format were coordinate-sorted and converted to BAM format using SAMTOOLS (version 0.1.18; Li et al., 2009). In this study, we reused the BAM files to search for structural variants that are potentially associated with obesity.

### Bioinformatic detection of structural variants

In order to detect SVs in short paired-end sequencing data, we used two bioinformatic methods: DELLY (Rausch et al., 2012) and TARDIS (Soylev et al., 2019). DELLY has the ability to detect deletions, inversions, duplications, and interchromosomal translocations with its integrated analysis of split reads. TARDIS is a more recent method with the ability to detect more complex events, such as a tandem duplication.

### Principal components analysis

Top principal components were included as covariates in the association analysis to account for population stratification. Principal components analysis (PCA) was conducted using plink software (ver. 2.0) on a sample of 50 sequenced dogs. We utilized 6.5 million short variants with a minor allele frequency (MAF) greater than 0.05.

### Selecting variants for droplet digital PCR

Due to the separate analysis of each genome by the two algorithms, the predicted localizations of structural variants (SVs) may vary across samples. To identify chromosomal regions potentially associated with obesity, we utilized the CoNVaQ algorithm (Larsen et al., 2018). In CoNVaQ, a new region is initiated whenever an SV from any dog starts or ends. This tool enables the comparison of two sets of SV segments, searching for genomic regions where the occurrence of SVs is significantly associated with the phenotype. In our study, we compared 28 obese dogs with 22 nonobese dogs.

We considered regions with a CoNVaQ raw *p*-value below 0.05 located within or in close proximity (<10 kb apart) to one or more protein-coding genes. CoNVaQ utilizes a straightforward statistical model that incorporates Fisher’s exact test, thereby excluding the consideration of other potential risk factors, such as sex, age, castration, and genotype at the *POMC* locus. The CoNVaQ output, however, includes the “Gain/Loss” status for each individual, facilitating the verification of associations in a statistical model that incorporates known risk factors.

The SV calls (Gain/Loss) obtained from CoNVaQ were used in the general linear model for body weight and the logistic regression model for obesity. These models also incorporated age (as a covariate), sex (male/female), neuter status (yes/no), the top three principal components, and the presence or absence of the *POMC* deletion (14-bp Del variant, as described by Raffan et al., 2016). With regard to the *POMC* gene, the homozygous deletion was observed in only two dogs, therefore the Del/Del dogs and heterozygotes were classified as a single group.

In order to narrow down the chromosome regions identified in the previous procedure and select regions for ddPCR assays, we compiled a list of genes associated with BMI in humans. To do this, we utilized the GWAS Catalog (www.ebi.ac.uk/gwas, accessed on 5 February 2022), which provided us with single-nucleotide polymorphisms (SNPs) reported in genome-wide association studies (GWAS) that demonstrated a significant association (*p* < 1.0 × 10^−9^) with BMI, along with their corresponding mapped genes. This comprehensive process resulted in a list of 1966 unique gene names.

### Droplet digital PCR for CNV analysis

DNA was extracted from frozen blood samples with MasterPure DNA Purification Kit for Blood Version II (Epicenter). The DNA quality was tested using a Qubit 2.0 Fluorometer (Invitrogen). Primers and assays for droplet digital PCR (ddPCR) of the fragments were designed by the tool on the BioRad website and by the Primer3PLus program. The finished primers and assays were obtained from BioRad or the Institute of Biochemistry and Biophysics of the Polish Academy of Sciences. A T100TM thermal cycler, a QX200TM droplet generator, a PX1TM PCR plate sealer, and a QX200TM droplet reader were obtained from BioRad. The reaction PCR was prepared using ddPCR Supermix for Probes (BioRad). The restriction enzymes (*Hae*III, *Eco*RI, *Msp*I, *Xba*I) were added to the reaction mix to improve the distribution of DNA across the droplets.

### Association analysis

To further explore the association between structural mutations identified by ddPCR and obesity-related traits in the sample of 110 dogs, we employed the general linear model (Type III SS) with body weight and logistic regression model with obesity status as the outcomes. These models incorporated age (as a covariate), sex, neuter status, presence of the *POMC* deletion (presence of 14-bp Del variant), and copy number determined by ddPCR (as a covariate). It is important to note that since principal components were not available for the unsequenced dogs, the association analysis was not adjusted for population stratification. Regression analyzes were performed using R statistical software version 4.2.2.

We adopted a significance level of alpha = 0.05, and the resulting *p*-values were left unadjusted. Regardless of whether multiple-test correction is implemented or not, a cohort consisting of 110 individuals inherently lacks the capacity to offer unequivocal statistical substantiation. Within a limited population, the correction of *p*-values for variants pre-selected with a relatively high *a priori* likelihood substantially diminishes the likelihood of identifying prospective associations.

## Results

### Bioinformatic detection of structural variants

Analysis of the paired short-read sequencing data allowed for initial identification of almost 400,000 structural variants within 50 dog genomes. It should be noted, however, that the percentage of false results for short-read-based methods may be extremely high. To ameliorate this problem, we discarded the 10% longest variants from each method and excluded all variants shorter than 30bp. The two algorithms differed significantly in the number and size of the variants they found: DELLY identified an average of 7,950 variants per dog (with a median size of 402bp) while TARDIS detected only 500 variants per dog (with a median size of 512bp). A summary of the results is presented in Table 2. Because most false positive variants are represented by a single individual, such variants are unlikely to be statistically associated with obesity.

**TABLE 2:** Summary of structural variants detected by bioinformatic analysis of paired short-read sequencing data. Ten percent of the largest variants from each method were excluded.

Type	DELLY			TARDIS		
	N/dog	Size range	Median size (bp)	N/dog	Size range	Median size (bp)
Deletion	7,054	33bp–207 kb	389	318	31bp–62 kb	461
Insertion	180	38bp–86bp	58	77	31bp–60 kb	93
Inversion	715	50bp–206 kb	2,561	2	40bp–60 kb	1,013
Tandem duplication	—	—	—	102	33bp–59 kb	3,205
**All**	7,950	33bp–207 kb	402	500	31bp–62 kb	512

We proceeded to use the CoNVaQ method to identify chromosomal regions that exhibited differences in structural variation (SV) frequency between obese and nonobese dogs. A total of 1,291 regions demonstrated a raw *p* below 0.05 (ranging from 7.7 × 10^−7^ to 4.7 × 10^−2^), with 241 of these regions overlapping with one or two protein-coding genes (Supplementary Figure S1). The SV calls (gain/loss) obtained from CoNVaQ were utilized in regression models for body weight and obesity, which included age, sex, neuter status, the top three principal components, and *POMC* deletion. The score plot for the first two principal components is shown on Supplementary Figure S2. The graph does not show an unambiguous picture of population stratification. Principal components and the effect of deletion in the *POMC* gene were not statistically significant, but were included in the models. Following the exclusion of regions with a *p* exceeding 0.05, a total of 56 regions remained (raw *p* ranging from 7.6 × 10^−5^ to 4.8 × 10^−2^).

Among the 56 regions that were analyzed, only one region corresponded to the list of GWAS signals associated with human BMI. To determine which SV variants should be further examined, we took into account both the results of the initial association analysis (considering low *p*) and the proximity of the variants to protein coding genes. After careful deliberation, we chose the following seven genes for additional molecular analysis: *ALPL*, *KCTD8*, *RYR3*, *SGM1*, *SLC12A6*, *VPS26C*, and *MME* (with corresponding *p* of 2.8 × 10^−3^, 3.1 × 10^−3^, 4.8 × 10^−4^, 2.8 × 10^−3^, 4.8 × 10^−4^, 2.9 × 10^−3^, and 1.4 × 10^−4^, respectively). It is worth noting that initially, four other genes (*RECQL4*, *RBP1*, *XRN1*, and *GTF2A16*) were also considered. However, designing assays for them proved to be impossible due to the presence of highly similar fragments in different parts of the reference genome. *RYR3* was selected due to its paralog, *RYR1*, which has been shown to influence the distribution of adipose tissue in gilts (Fujii et al., 1991; Sadkowski et al., 2015).

### Results of ddPCR and association study

**FIGURE 1:**
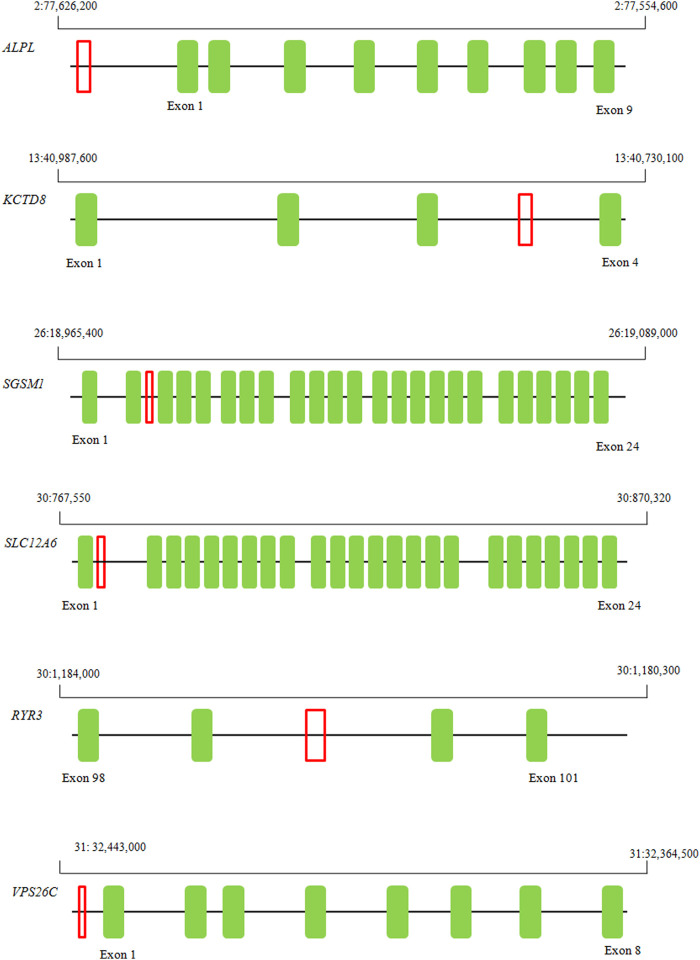
Localization of six ddPCR-amplified fragments exhibiting overlap with structural variants that hold potential implications for body weight and obesity susceptibility in Labrador.

The assays were successfully designed for seven fragments. We confirmed the presence of structural polymorphism in six genes: *ALPL, KCTD8, SGSM1, SLC12A6*, *RYR3,* and *VPS26C*, though dd-PCR showed no signal of structural variants for the *MME* gene. The chromosomal location of the six amplified fragments are presented on Figure 1.

**FIGURE 2:**
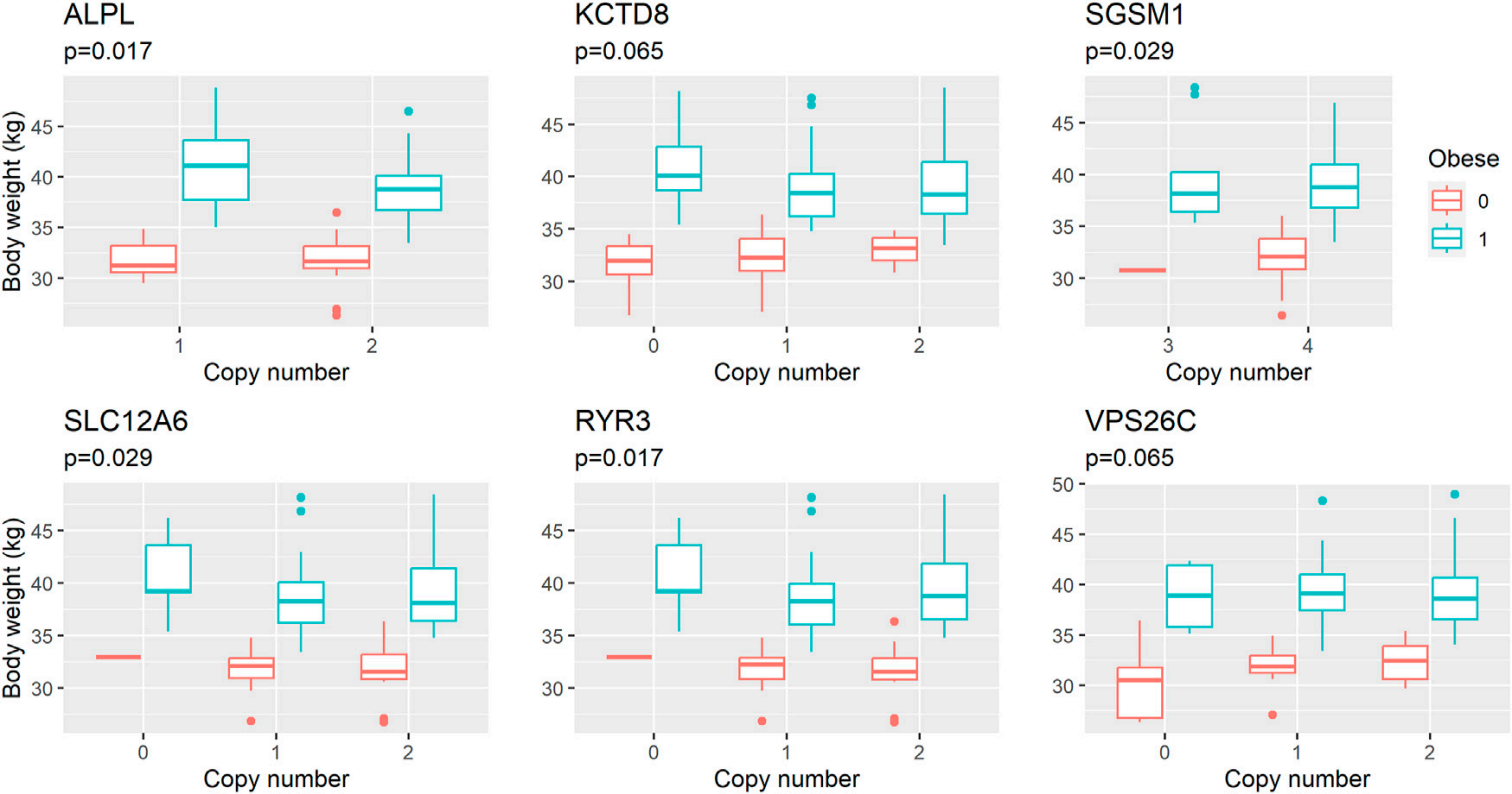
Composite boxplot illustrating the relationship between body weight and copy number across six distinct genes. Each boxplot within the composition showcases the distribution of body weight values associated with varying copy number states for a specific gene. The median body weight within each gene’s copy number category is indicated by the central line within the box, with the top and bottom edges of the box representing the third and first quartiles, respectively. Whiskers extend to 1.5 times the interquartile range (IQR) from the box boundaries, encompassing the majority of data points. Outliers, depicted as individual data points, are situated beyond the whiskers. The *Y*-axis represents body weight (grams), while the *X*-axis designates the six different genes and their respective copy number states. The *Y*-axis represents body weight (in kilograms), corrected by age (as a covariate), sex, neuter status and presence of the *POMC* deletion (presence of 14-bp Del variant). Owing to the limited size of certain groups, they are omitted from the plot for clarity. *p*-values (*p*) refer to the effects of polymorphisms estimated in linear regression model.

**FIGURE 3:**
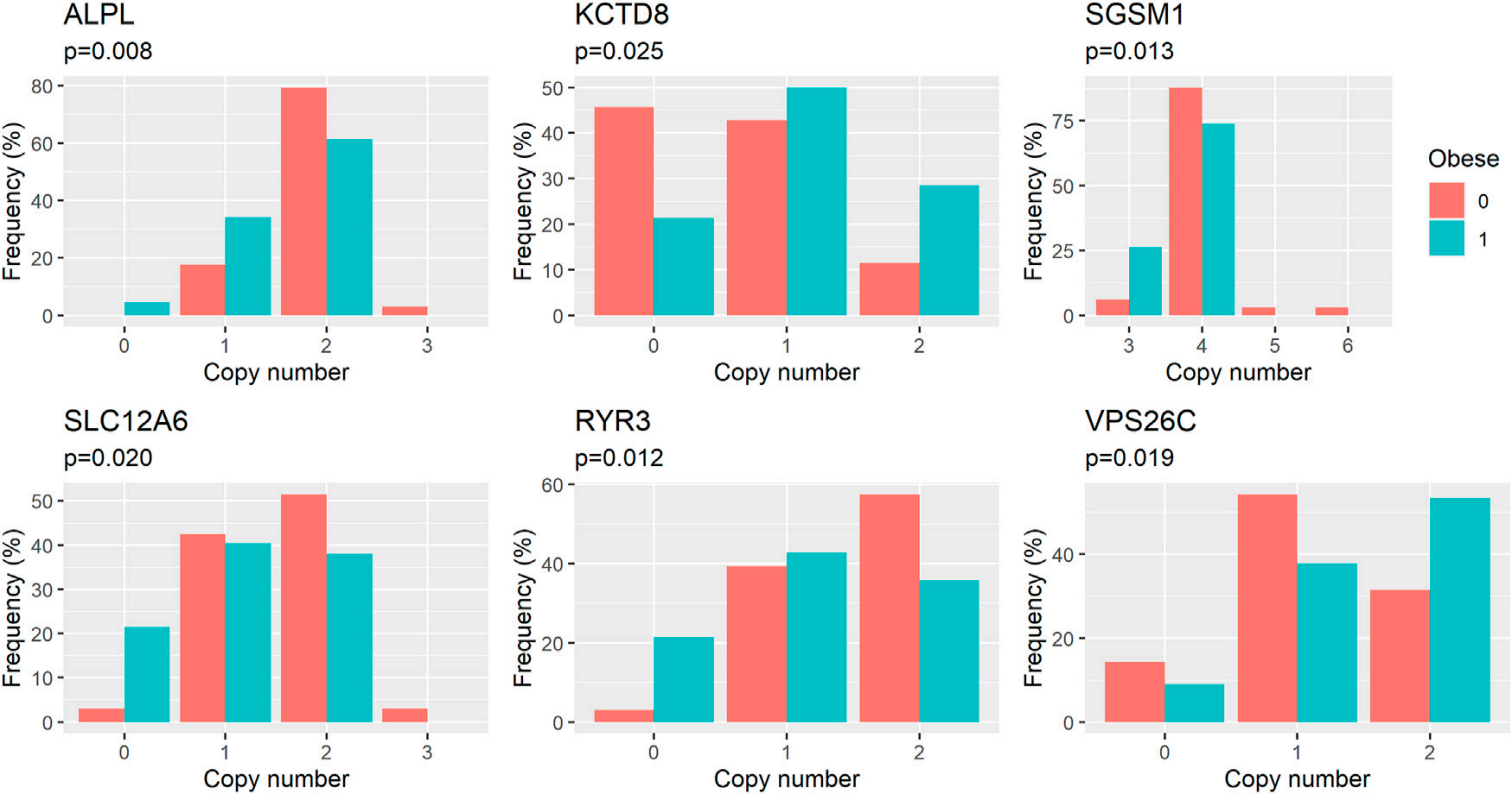
Distribution of genotypes (here identified by the number of copies of a DNA fragment in the genome) in groups of obese and healthy dogs. *p*-values (*p*) refer to the effect of copy number on the risk of obesity as estimated in logistic regression model.

**FIGURE 4:**
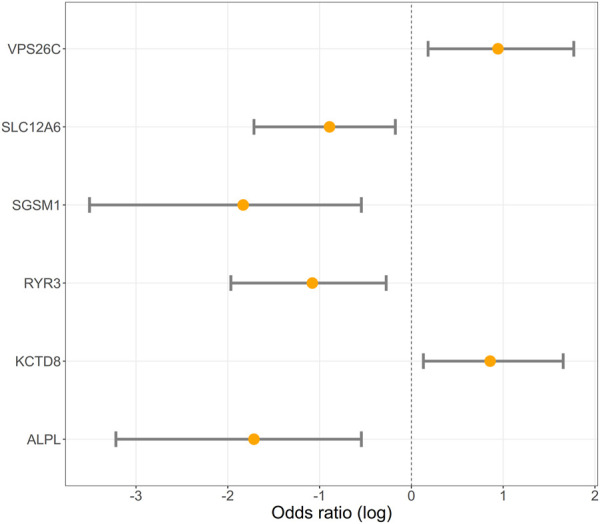
Logarithmic scale illustration of odds ratios (OR) with 95% confidence intervals (CI) for six distinct genes. The *X*-axis denotes the logarithm of odds ratios, where a value of 0 signifies no effect. Values greater than zero mean that the risk of obesity increases with the number of copies of the DNA fragment. Each gene’s odds ratio is represented by a point estimate on the log scale, accompanied by a 95% CI illustrated as an error bar. The inclusion of confidence intervals offers insights into the precision and significance of the odds ratio estimates.

We performed an association analysis between ddPCR results and obesity characteristics observed in the group of 110 Labrador. Again, the effect of deletions in the *POMC* gene was not statistically significant, but was included in the models. The structural variants observed in six genes exhibited associations with obesity, with four out of the six genes showing associations with both body weight and obesity (unadjusted *p* = 0.03, as shown in Table 3 and Figure 2; Figure 3; Figure 4). The effects of the four statistically significant mutations on body weight ranged from 34% to 54% SD. Notably, the results remain consistent for both traits, indicating that variants associated with increased body weight also elevate the risk of developing obesity.

**TABLE 3:** Results of association study between body weight and obesity and structural variants in six protein-coding genes.

Localization^a^	Range of ddPCR output^b^	Association	
		Body weight (kg)^c^ (per copy ±SE)	Obesity^d^ (odds ratio)
2:77,622,662–77,622,741 deletion, intergenic region between ***ALPL*** and *ECE1*	0–2.5	−2.88 ± 1.17 *p* = **0.017**	OR = 0.18 95%CI: 0.04–0.58 *p* = **0.008**
13:40,904,429–40,904,512 deletion, intron 3 of ***KCTD8***	0–2.1	1.55 ± 0.83 *p* = 0.065	OR = 2.36 95%CI: 1.14–5.23 *p* = **0.025**
26:18,996,505–18,996,597 duplication, intron 1 or 2 of ***SGSM1***	2.8–5.8	−2.68 ± 1.20 *p* = **0.029**	OR = 0.16 95%CI: 0.03–0.58 *p* = **0.013**
30:774,403–774,537 deletion/duplication*,* intron 1 of ***SLC12A6***	0–3.3	−1.84 ± 0.82 *p* = **0.029**	OR = 0.41 95%CI: 0.18–0.84 *p* = **0.020**
30:1,182,396–1,182,519 deletion, intron 99 of ***RYR3***	0–2.1	−2.17 ± 0.88 *p* = **0.017**	OR = 0.34 95%CI: 0.14–0.76 *p* = **0.012**
31:32,435,989–32,436,088 deletion, intergenic region between ***VPS26C*** and *DYRK1A*	0–2.2	1.64 ± 0.87 *p* = 0.065	OR = 2.57 95%CI: 1.20–5.87 *p* = **0.019**

^a^ ddPCR, amplified fragment (CanFam 3.1). The true chromosomal location of the polymorphism is unknown, but it covers the amplified fragment.

^b^ Values obtained from ddPCR, are real numbers and approximate the number of copies of the variant in the genome.

^c^ Estimated effect in kg per copy and the corresponding standard error as estimated in general linear model.

^d^ Odds ratio (OR) and the corresponding 95% confidence interval (95%CI) as estimated in logistic regression for binary trait. Odds ratio = 1 indicates no difference between the groups.

Values <0.05 are highlighted in bold.

The findings for the *RYR3* and *SLC12A6* genes are particularly intriguing, as these genes are closely located on chromosome 30, and their genotype distributions displayed remarkable similarity, suggesting a strong linkage disequilibrium. In the case of the *RYR3* gene, we amplified a 124-bp fragment located in intron 99 (CanFam3.1). The number of copies of this mutation ranged from zero to two, with a mean of 1.6 for dogs with a body condition score (BCS) of 3, and only 1.0 for those with a BCS of 5. This suggests that dogs carrying a deletion at the *RYR3* gene are more predisposed to excessive fat accumulation, while the risk of obesity decreases with each copy of the reference variant. Similarly, the deletion within the SLC12A6 gene increases the risk of obesity. Collectively, the results pertaining to the *RYR3* and *SLC12A6* genes provide evidence of the potential importance of the chromosome 30 region in canine obesity, warranting further investigation.

The aforementioned associations were not adjusted for population stratification. In the preceding step, which encompassed fifty sequenced dogs, we computed the top twenty principal components. The eigenvalues for these components ranged from 2.6 to 1.0. The score plot for the first two principal components indicated genetic homogeneity within the population (Supplementary Figure S1). We believe that the procedure used here would reveal hidden stratification of the population, including that resulting from two types of Labrador (American and English).

## Discussion

The genetics of canine obesity is in its infancy compared to the genetics of human obesity, but for this reason, human genetics can offer a number of valid hypotheses, the verification of which in the canine population has recently brought significant progress. A number of candidate gene studies in relatively small cohorts have identified short mutations in the dog genome that shape traits associated with obesity in various breeds. A 14-bp intronic deletion in the *POMC* gene greatly increases the body mass of Labrador retriever dogs, while altering the function of β-MSH and β-endorphin, two peptide hormones associated with food motivation and adiposity (Raffan et al., 2016; Mankowska et al., 2017). Moreover, two SNPs in the noncoding parts of *TNF* gene have been shown to be important for polygenic obesity in Labrador retriever dogs (Mankowska et al., 2016), whereas two exonic SNPs in the *MC4R* gene have been linked to body weight in Beagle dogs (Zeng et al., 2014). These findings indicate that the contribution of single genes to polygenic obesity may be greater in dogs than in humans, and suggests the possibility of identifying key markers that would be useful in risk prediction and treatment of obesity without the need for expensive genome-wide profiling.

In this study, our focus was on structural polymorphisms in the dog genome. Generally, structural polymorphisms are less prevalent than short variants, but their potential impacts can be anticipated to be relatively significant and easier to detect in small populations. While copy number variations (CNVs) have not been as extensively investigated as short variants, there is compelling evidence linking certain CNVs to BMI in humans. For instance, such variants have been identified near the *NEGR1*, *GPRC5B*, and *PPYR1* genes (Willer et al., 2009; Speliotes et al., 2010; Jarick et al., 2011). Additionally, the 1p21.1 multi-allele CNV encompasses *AMY1A*, which encodes salivary α-amylase.

We did not find any evidence linking dog obesity traits to structural variants in or near genes that are considered crucial for obesity development, such as genes involved in the leptin-melanocortin signalling pathway and genes important for neuronal circuitry development (e.g., *BDNF*, *NTRK2*, *SEMA3A-G*). In human and animal models, these genes often harbour rare mutations that are responsible for severe and early-onset obesity. Furthermore, it is increasingly recognized that variants in or near these genes influence the risk of common polygenic obesity in humans. Despite the dominance of studies on these genes in the field of obesity genetics, it appears unlikely that these genes alone account for a significant portion of the genetic variation in obesity. In human studies, genome-wide association studies (GWAS) for obesity-related traits have identified thousands of loci, the majority of which have unknown underlying mechanisms. Our research, limited to common structural variants, suggests that these variants may be involved in mechanisms that have yet to be understood.

We obtained preliminary evidence suggesting that variants in six genes warrant further investigation. In our study, a structural deletion near the *ALPL* (Alkaline Phosphatase, Biomineralization Associated) gene was associated with reduced body weight and a higher risk of obesity. In humans, mutations in this gene have been linked to hypophosphatasia, an inherited condition that disrupts bone and teeth mineralization. Moreover, it is intriguing that this gene has also been associated with adaptive thermogenesis, which has garnered significant attention for its role in increasing systemic energy expenditure and combating obesity and diabetes. In mice, the targeted removal of *ALPL* specifically in adipocytes leads to decreased overall energy expenditure and the onset of obesity, without affecting movement or feeding behavior (Sun et al., 2021). These findings highlight the potential importance of *ALPL* in regulating energy balance and adiposity.

We have obtained statistical evidence suggesting that a structural deletion in or near the *KCTD8* (Potassium Channel Tetramerization Domain Containing 8) gene could potentially increase body condition score. Additionally, it has been reported that an SNP in the Arabic population may be linked to diabetes (Musambil and Siddiqui, 2019). While clear associations between this gene and obesity traits are lacking, it is of interest due to its role in bitter and sweet taste signaling pathways. The gene is particularly highly expressed in the brain, but lower expression levels have also been observed in adipose tissue.

Our data also revealed that a duplication in the first intron of the *SGMS1* (Small G Protein Signaling Modulator 1) gene may lead to reduced body weight and a lower risk of obesity. The *SGSM1* gene is involved in GTPase activator activity and small GTPase binding activity, and it exhibits high expression levels in the brain. Interestingly, an intron variant in this gene has been associated with insulin measurement (Julienne et al., 2020).

We have demonstrated that deletions in or near the *SLC12A6* (Solute Carrier Family 12 Member 6) and *RYR3* (Ryanodine Receptor 3) genes are statistically associated with decreased body weight and a lower risk of obesity. These two genes are located in close proximity to each other on chromosome 30, and their effects on the two obesity traits are similar. In humans, mutations in the *SLC12A6* gene are generally associated with neuropathy (Bogdanova-Mihaylova et al., 2021), but there is no known connection between mutations in this gene and obesity-related traits, except for one genome-wide association study signal for BMI (Dong et al., 2018). Interestingly, the *SLC12A6* gene has been studied in both humans and dogs, revealing that truncating variants are responsible for movement disorders, although the phenotypes differ between species (Van Poucke et al., 2019).

The ryanodine receptor, encoded by the *RYR3* gene, plays a crucial role in releasing calcium from intracellular storage for various cellular processes, particularly in skeletal muscle contraction. It has been found that *RYR3* is under strong selection in sport-hunting genes (Kim et al., 2018). In humans, a single nucleotide polymorphism of this gene has been correlated with diabetes and hypertension, diseases associated with obesity (Gong et al., 2018). Genome-wide linkage analysis studies have also demonstrated a connection between polymorphism in the *RYR3* gene and the function of adiponectin in a Chinese population (Chang et al., 2016). In pigs, a short mutation in the *RYR1* gene, which is part of the intracellular calcium ion channel family like *RYR3*, is known to affect meat quality and fatness traits (Sadkowski et al., 2015). Additionally, an intronic variant in the human *RYR3* gene has been associated with hippocampal volume (Alliey-Rodriguez et al., 2019). Studies on rats have shown that obesity is associated with abnormal neuronal differentiation in the hippocampus (Liśkiewicz et al., 2021).

We also observed an association between a deletion near or in the *VPS26C* (VPS26 Endosomal Protein Sorting Factor C) gene and an increased risk of obesity. However, we are not aware of any studies that link this gene specifically to obesity.

In our studies, we did not observe any impact of the known deletion in the *POMC* gene on body weight or obesity. The reason behind our failure to detect the effect of the *POMC* gene may be attributed to the rare occurrence of the deleterious variant in the homozygous genotype. It is plausible to assume that this deletion, which significantly disrupts gene function, follows a recessive pattern, thereby explaining our findings. We included the effect of *POMC* in all of our statistical models as a precautionary measure. In populations of limited size that lack substantial statistical power to detect significant sources of variation, incorporating a factor based solely on statistical significance can potentially lead to inaccuracies. Consequently, the inclusion of the *POMC* gene in the model was guided by existing knowledge that establishes its significance as a source of variation in Labrador.

Our association study is predicated upon a comparatively modest population, wherein a majority of individuals have not undergone sequencing. The extensive repertoire of association studies conducted within the human population has established that, owing to the vastness of the genome and the inherent role of chance, investigations involving small populations are particularly susceptible to yielding false positive outcomes. Although the mitigation of spurious associations is feasible through adjustments for both multiple testing and population stratification, these safeguards were not applicable in the context of our study. As alluded to earlier, a significant portion of individuals lacked complete genome screening, thus impeding a comprehensive accounting for potential population stratification. Given the constraints of the small sample size and the inherent limitations of a singular test, no provisions were made for multiple testing adjustments. Consequently, our findings should be regarded as moderately indicative statistical evidence that does not definitively resolve the matter of association but holds potential to inform and direct subsequent investigations.

Our research did not include a functional analysis, nor did we attempt to explain the mechanisms underlying the statistical associations described here. We acknowledge that elucidating such mechanisms will be challenging, particularly because the genes identified as important for Labrador obesity traits in our research have not been studied in the context of human obesity thus far. However, irrespective of the underlying mechanism, confirming the statistical relationship between the identified structural variants and obesity can serve as an indirect objective in the breeding of Labrador.

In conclusion, we have provided statistical evidence suggesting that common structural mutations in or near six genes, namely, *ALPL*, *KCTD8*, *SGSM1*, *SLC12A6*, *RYR3*, and *VPS26C*, may play a role in the variability of body weight and body condition score in Labrador retriever dogs. These findings emphasize the need for additional research to validate the associations.

## Data Availability

Publicly available datasets were analyzed in this study. This data can be found here: European Nucleotide Archive (PRJEB47658).

## Supplementary material

SUPPLEMENTARY FIGURE S1 Manhattan plot for chromosomal regions potentially associated with obesity in Labrador retrievers. P-values are derived from the comparison of the frequency of structural variants between the groups of obese and healthy dogs using the CoNVaQ tool. Only 1291 regions with p-values>0.05 are shown. Chromosome 39 represents the X chromosome.

SUPPLEMENTARY FIGURE S2 The score plot for the first two principal components for the sample of 50 dogs.
