# Supplementary figures and images for: Uncovering structural variants associated with body weight and obesity risk in labrador retrievers: a genome-wide study
Source: Front Genet. 2023 Sep 20;14:1235821. doi: 10.3389/fgene.2023.1235821 (PMC10548226; doi:10.3389/fgene.2023.1235821)

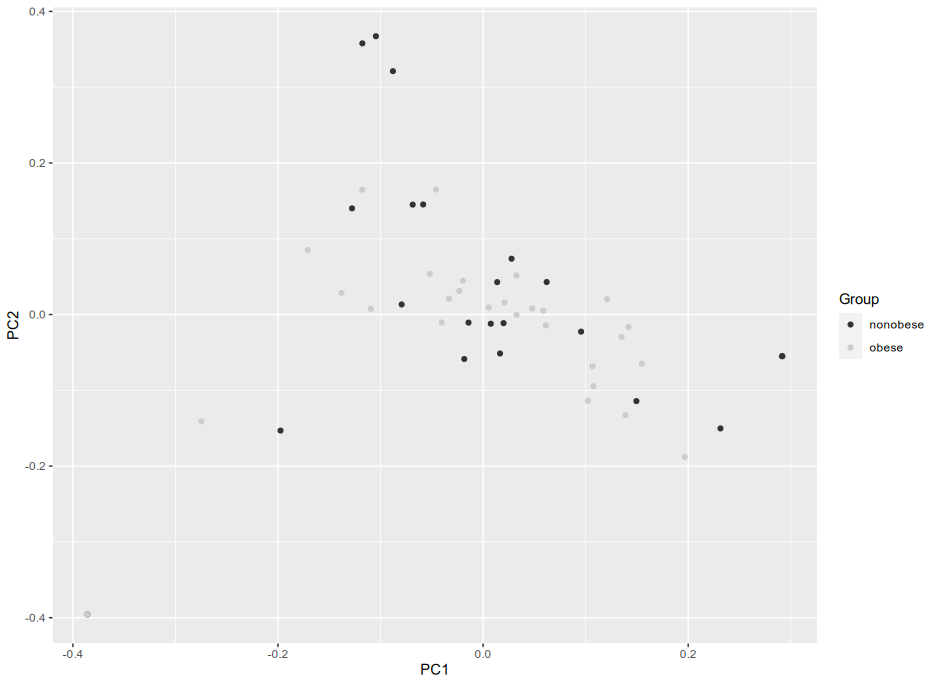

Supplement: Supplementary file 1 [file Image2.TIF]

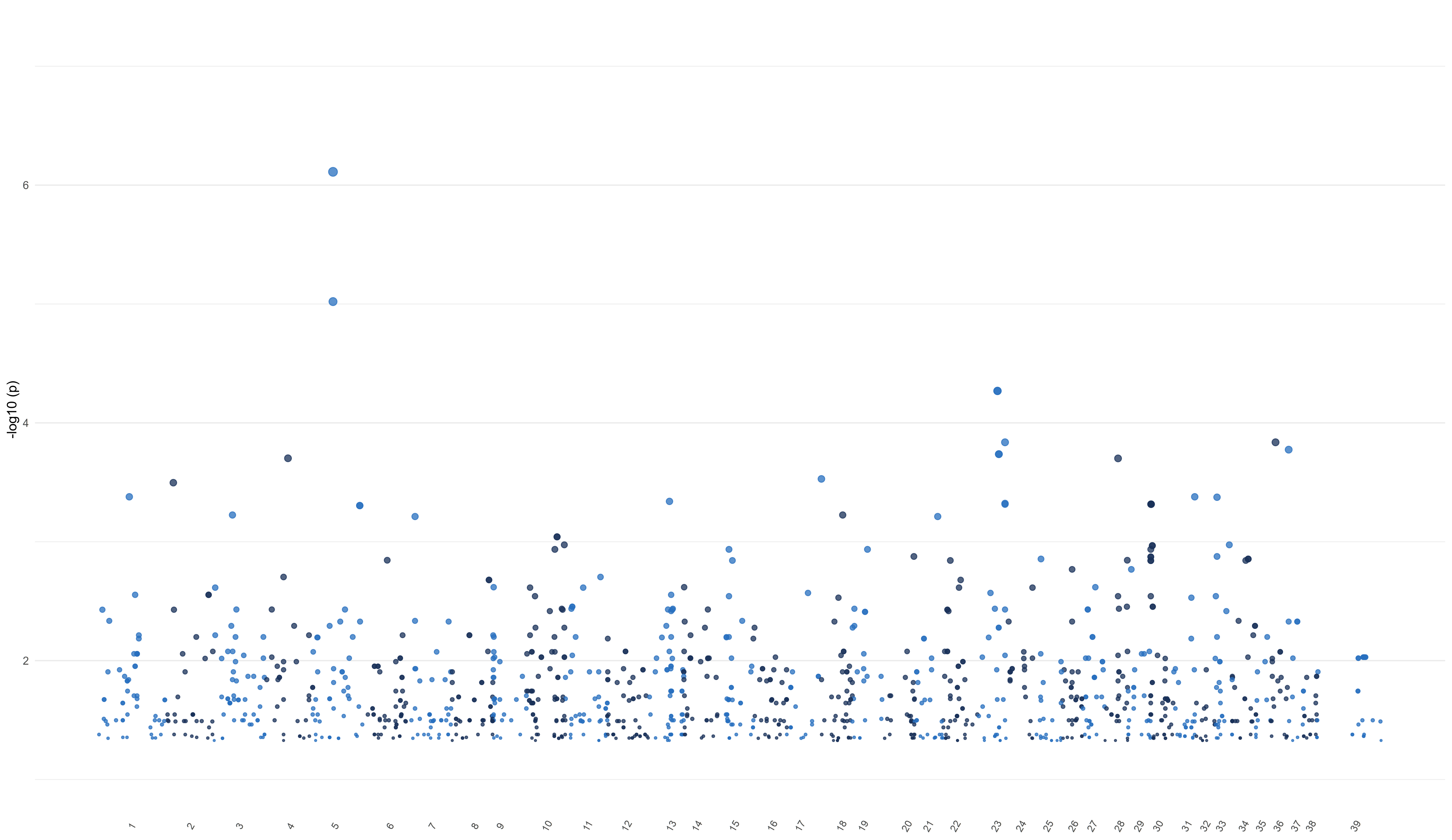

Supplement: Supplementary file 2 [file Image1.TIF]
